# Vertebral fracture in elderly female patients after posterior fusion with pedicle screw fixation for degenerative lumbar pathology: a retrospective cohort study

**DOI:** 10.1186/s12891-019-2534-z

**Published:** 2019-05-29

**Authors:** Masahiro Nakahashi, Hiroshi Uei, Yasuaki Tokuhashi, Masafumi Maseda, Hirokatsu Sawada, Hirotoki Soma, Hiroyuki Miyakata

**Affiliations:** Department of Orthopaedic Surgery, Nihon University School of Medicine, 30-1 Oyaguchi Kami-cho, Itabashi-ku, Tokyo, 173-8610 Japan

**Keywords:** Vertebral fracture, Elderly female, Pedicle screw fixation, Complication, Osteoporosis, Dual-energy X-ray absorptiometry

## Abstract

**Background:**

There have been only a few reports of subsequent postoperative vertebral fracture following posterior spinal instrumentation fusion, especially in elderly female patients. This study attempted to evaluate the long-term prevalence of subsequent postoperative vertebral fracture in female patients aged 70 years and older who underwent spine decompression and fusion surgery with pedicle screw fixation.

**Methods:**

We retrospectively reviewed prospectively collected data from 125 patients who met our inclusion and exclusion criteria. Patients were divided into 2 groups according to age: patients aged 70 years and older (Group A) and patients aged under 70 years of age (Group B). We evaluated incidence of subsequent postoperative vertebral fractures, type and timing of vertebral fractures, preoperative bone mineral density (BMD), preoperative diagnosis, surgical procedure, number of levels fused, extension of fusion to the lumbosacral junction, and presence of a transverse fixator.

**Results:**

Baseline characteristics excluding patients’ age were not statistically different between the two groups. Preoperative BMD of Group A was an average 81.7% of the young adult mean (YAM) value and that of Group B was an average 85.1% YAM value. Subsequent postoperative vertebral fractures occurred in 22 (41.5%) of 53 in Group A. On the other hand, fracture occurred in 17 (23.6%) of 72 in Group B. There were significant differences between the groups (*p* = 0.02). The odds ratio for subsequent vertebral fracture was 2.4 (95% confidence interval: 1.1–5.2) in favor of Group A. Survival analysis showed that the rate of subsequent vertebral fracture was significantly higher in Group A (log-rank test, *P* = 0.007).

**Conclusions:**

The incidence of subsequent vertebral fracture in patients aged 70 years and older was significantly higher than in patients aged under 70 years of age. In the case of pedicle screw fixation in elderly female patients, it is necessary to note the high risk of subsequent vertebral fracture despite short or non-rigid fusion. Vertebral fracture after posterior fusion surgery even for degenerative lumbar pathology could occur in more than one-third of female patients aged 70 years and older.

## Background

Recently, posterior spinal instrumentation fusion with segmental pedicle screws has been widely applied for the treatment of degenerative spine diseases in elderly patients. On the other hand, it has been recognized that adjacent fused disc problem is caused by rigid pedicle screw fixation [1–6]. The rates of revision surgery due to proximal junctional kyphosis/failure (PJK/PJF) following correction and fusion surgery for adult spinal deformity (ASD) is high, and Osteoporosis are risk factors of PJK/PJF following ASD surgery [7, 8]. However, there have been only a few reports of postoperative spinal vertebral fracture following posterior spinal fusion surgery for degenerative lumbar pathology excluding ASD, and even fewer reports of the effects of pedicle screw fixation on fixed vertebrae. This study attempted to evaluate the long-term prevalence of subsequent postoperative vertebral fracture in female patients aged 70 years and older who underwent spine decompression and fusion surgery with pedicle screw fixation.

## Methods

### Patient population

This study was a retrospective review of a prospectively collected data from 2286 patients who underwent spine surgery at our institution between 1997 and 2006. Those patients included for analysis were postmenopausal female patients and had a primary diagnosis of degenerative lumbar pathology with instability and required posterior spinal fusion with pedicle screw constructs. Patients were excluded if they had vertebral compression fractures, posttraumatic kyphosis, metastatic spinal tumors, myelomas, spinal infections, metabolic bone diseases such as osteomalacia and hyperparathyroidism, or required greater than 4-level fusion for realignment of spinal deformity due to adult spinal deformity. The study protocol was approved by Nihon University Hospitals’ Joint Institutional Review Board. All participants provided written informed consent. Finally, 125 patients were enrolled in this study. In our cohorts, patients were divided into 2 groups according to age: patients aged 70 years and older (Group A) and patients aged under 70 years of age (Group B).

### Outcome evaluation

We evaluated the incidence of subsequent postoperative vertebral fractures, and the type and timing of vertebral fractures. The types of subsequent vertebral fracture were classified into three types according to the relationship between fusion level and fracture level: adjacent fused level, remote level, and instrumented level. Furthermore, we evaluated preoperative diagnosis, preoperative bone mineral density (BMD), surgical procedure, number of levels fused, extension of fusion to the lumbosacral junction, and presence of a transverse fixator. Lateral spine radiographs obtained at before surgery, at 3 and 6 months after surgery, and yearly after surgery to the final follow-up were used for morphometric vertebral fracture ascertainment. Asymptomatic subsequent vertebral fractures were defined as a new fracture with a decrease of more than 20% in any vertebral height from baseline [9]. Diagnostic criteria of symptomatic subsequent vertebral fractures were as follows: acute increase in back pain as a result of a fall from standing height or less or without any trauma, radiologic evidence of acute vertebral fractures determined by magnetic resonance imaging, showing geographic patterns of low-intensity-signal changes on T1-weighted images, and high-intensity-signal changes on T2-weighed images [10]. Dual Energy X-ray Absorptiometry® (HOLOGIC, Bedford, MA) of the lumbar spine (L2-L4) was also performed to evaluate preoperative BMD.

### Statistical analysis

The data were analyzed using SPSS 19.0 (SPSS Inc., Chicago, IL). The distribution of the variables was expressed as the mean standard deviation and range. Paired *t* test, Mann-Whitney U test, or χ^2^ test were used for comparisons between the two groups. Cumulative incidences of the subsequent postoperative vertebral fractures were estimated by the Kaplan-Meier method, and differences between groups were assessed with the log-rank test. *P*-values < 0.05 were considered statistically significant.

## Results

Group A consisted of fifty-three patients, aged 70 years and older (70–86 years, average 74.2 years). Group B consisted seventy-two patients under 70 years of age (50–69 years, average 61.7 years) (Table 1). In Group A, preoperative diagnoses were degenerative spondylolisthesis in 26 (49.0%), spinal stenosis in 26 (49.0%), and spondylotic spondylolisthesis in 1 (1.9%). In Group B, preoperative diagnoses were degenerative spondylolisthesis in 38 (52.8%), spinal stenosis in 31 (43.0%), and spondylotic spondylolisthesis in 3 (4.2%). The BMD of Group A was an average 81.7% of the young adult mean (YAM) value and that of Group B was an average 85.1% YAM value. There was no significant difference in BMD YAM value between the groups (*P* = 0.32). The surgical procedures for Group A and Group B were posterior lumbar interbody fusion (PLIF): 18 (33.9%) and 24 cases (33.3%), posterolateral fusion (PLF): 12 (22.6%) and 19 (26.4%) cases, and PLF combined with PLIF: 23 (43.4%) and 29 (40.3%) cases, respectively. The mean number of levels fused for Group A and Group B were 2.2 and 2.1, respectively (*P* = 0.37). Regarding the types of fusion length for Group A and Group B, the numbers of short-segment fusion (1 or 2 levels) were 32 (60.3%) and 49 (68.0%), and the numbers of long-segment fusion (3 or 4 levels) were 21 (39.6%) and 23 (31.9%), respectively (*P* = 0.37). The numbers of extension of fusion to the lumbosacral junction were 14 (26.4%) and 10 (19.4%), respectively (*P* = 0.35). The numbers of use of a transverse fixator were 22 (41.5%) and 23 (31.9%), respectively (*P* = 0.27).

**Table 1:** Baseline characteristics

Characteristic	group A (*n* = 53)	group B (*n* = 72)	*P* value
Age at surgery, mean (SD), years	74.2 (4.2)	61.7 (5.5)	< 0.001
Diagnosis, n (%)			0.66
Degenerative spondylolisthesis	26 (49.0)	38 (52.8)	
Spinal stenosis	26 (49.0)	31 (43.0)	
Spondylotic spondylolisthesis	1 (1.9)	3 (4.2)	
BMD YAM, mean (SD), %	81.7 (16.3)	85.1 (16.9)	0.32
Surgical Procedure, n (%)			0.88
PLIF	18 (33.9)	24 (33.3)	
PLF	12 (22.6)	19 (26.4)	
PLF combined with PLIF	23 (43.4)	29 (40.3)	
Number of levels fused, mean (SD), n	2.2 (0.9)	2.1 (0.9)	0.37
Types of fusion length, n (%)			0.37
short-segment fusion (1 or 2 levels)	32 (60.3)	49 (68.0)	
long-segment fusion (3 or 4 levels)	21 (39.6)	23 (31.9)	
Fusion to lumbosacral junction, n (%)	14 (26.4)	10 (19.4)	0.35
Use of a transverse fixator	22 (41.5)	23 (31.9)	0.27

*BMD indicates bone mineral density; YAM, young adult mean; PLIF indicates posterior lumbar interbody fusion; PLF, posterolateral fusion*

**Fig. 1:**
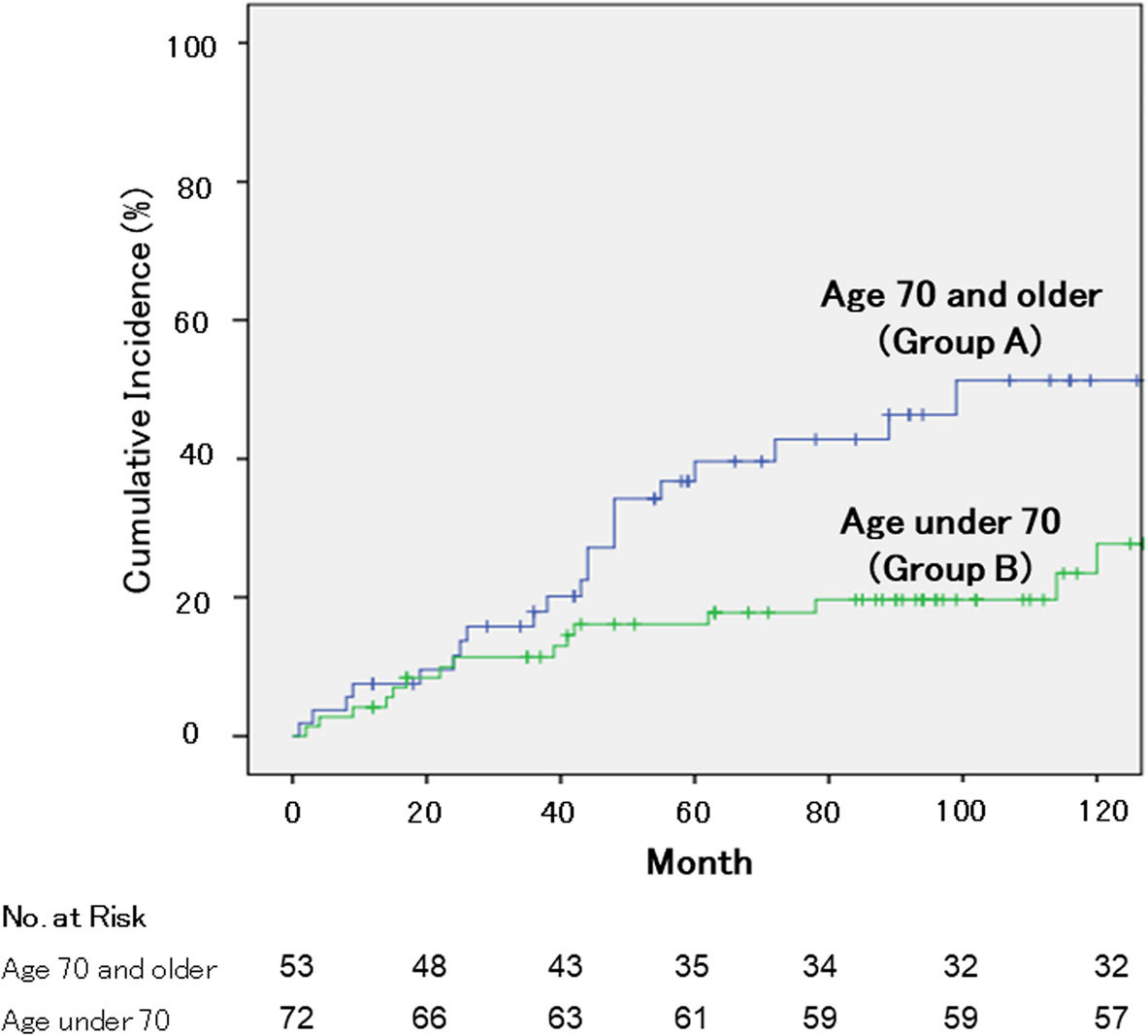
Incidence of subsequent vertebral fractures in each group. Survival analysis showed that the rate of subsequent vertebral fracture was significantly higher in Group A (log-rank test, *P* = 0.007)

The follow-up periods ranged from 12 to 151 months (average 76.8 months) for Group A, and 12–218 months (average 103.5 months) for Group B (*P* = 0.002) (Table 2). Subsequent postoperative vertebral fractures occurred in 22 (41.5%) in Group A and 17 (23.6%) in Group B. There were significant differences between the two groups (*p* = 0.02). The odds ratio of subsequent postoperative vertebral fracture between the groups was 2.4 (95% confidence interval: 1.1–5.2). The number of cases with each fracture type in Group A and Group B was as follows; adjacent level in 7 (31.8%) and 6 (35.3%), remote level in 8 (36.3%) and 7 (41.1%), and instrumented level in 7 (31.8%) and 4 (23.5%), respectively. Survival analysis showed that the rate of subsequent vertebral fracture was significantly higher in Group A than those in Group B (log-rank test, *P* = 0.007; Fig. 1).

**Table 2:** Comparison of postoperative clinical results in group A and group B

Parameter	group A (*n* = 53)	group B (*n* = 72)	*P* value
Follow up, mean (SD), (months)	76.8 (40.5)	103.5 (51.0)	0.002
Postoperative vertebral fracture, n (%)	22 (41.5)	17 (23.6)	0.02
Types of vertebral fracture, n (%)			0.18
adjacent level	7 (31.8)	6 (35.3)	
remote level	8 (36.3)	7 (41.1)	
instrumented level	7 (31.8)	4 (23.5)

## Discussion

It has been recognized that spinal fusion with pedicle screw fixation may cause adjacent-segment problems due to its rigidity [1–6]. Postoperative vertebral fractures with pedicle screw fixation are also relatively common complications. However, there are only a few published reports on its pathology.

Toyone et al. reported that 14 (24%) of the 59 female patients aged 55 years or older who underwent spinal fusion surgery with instrumentation for less than 4 segments had acute postoperative vertebral fracture [11]. Li et al. retrospectively reviewed 1936 patients who underwent instrumented spinal fusion surgery and were followed-up for at least three years [12]. They reported that subsequent vertebral compression fractures occurred in 224 patients (11.6%). They also investigated subsequent vertebral compression fractures after instrumented spinal fusion surgery (*n* = 6949) or non-fusion spinal surgery (*n* = 69,490) from the National Health Insurance Research Database of Taiwan [13]. They reported that the incidences of developing subsequent vertebral compression fractures were 130 patients (1.87%) for instrumented spinal fusion surgery and 175 patients (0.25%) for non-fusion spinal surgery, respectively. They reported that it was quite different from that found in previous studies [11, 12]. They concluded that the diagnosis of new subsequent vertebral compression fractures after spinal fusion surgery may be underestimated during outpatient follow-up because the insurance data did not provide detailed information. Our study demonstrated that subsequent postoperative vertebral fractures occurred in 22 (41.5%) in Group A and 17 (23.6%) in Group B from a retrospective review of a prospectively collected data. Long-segment fusion in adult spinal deformity surgery tended to have a higher cranial fracture rate since sagittal alignment of the spine is often corrected largely [8]. However, we excluded those who required greater than 4-level fusion for realignment of spinal deformity due to adult spinal deformity. Thus, vertebral fracture after posterior fusion surgery even for degenerative lumbar pathology could occur in more than 40% of female patients aged 70 years and older.

Lee et al. investigated the biomechanical effect of spinal fusion on the fused and adjacent segments [14]. They compared the rigidity of three different types of lumbosacral fusions. They concluded that all types of fusion produced increased stress on the adjacent unfused segments, with posterior fusion providing the greatest effect on the adjacent segments, and bilateral fusion being better than anterior fusion because bilateral fusion produced a lesser change in the axial stiffness. Chow et al. investigated the biomechanical effects of single level L4–5 and double L4-L5-S1 anterior interbody fusion on the adjacent unfused segments in cadaveric lumbar spine [15]. They reported that the segmental mobility at L2–3 and the intradiscal pressures in L2–3 after L4-L5-S1 fusion were significantly larger than those after L4–5 fusion in flexion. Our study demonstrated that the types of vertebral fracture for Group A and Group B were adjacent level fracture in 7 (31.8%) and 6 (35.3%), remote level fracture in 8 (36.3%) and 7 (41.1%), and instrumented level fracture in 7 (31.8%) and 4 (23.5%), respectively. There is a possibility that not only adjacent level fracture, but also remote level fracture and instrumented level fracture could occur after posterior fusion surgery in elderly female patients.

Bogdanffy et al. reported that patients who underwent combined anteroposterior fusion at L4-S1 exhibited decreased BMD at the L3 vertebral body at 3 and 6 months postoperatively. They concluded that this change could have been related to immobilization or altered biomechanics resulting from arthrodesis [16]. Lee et al. investigated that patients’ BMD of the vertebral bodies at the fused and at the unfused level following posterolateral fusion surgery with pedicle screw [17]. They concluded that the vertebral bodies at fused level may undergo osteoporosis in a pattern that is similar to what naturally occurs in the vertebral bodies at the unfused level. Myers et al. studied vertebral BMD in 8 patients who had instrumented lumbar fusion and in 8 matched control patients who had lumbar surgery with no fusion [18]. They concluded that patients who had undergone instrumented posterolateral lumbar fusions had decreased vertebral BMD at the level of fusion. Singh et al. studied 7 patients who underwent posterior lumbar instrumentation surgery at a mean 10.8-year follow-up. They concluded that lumbar BMD decreased gradually in vertebral levels with increased distance from the level of instrumented fusion [19]. Postmenopausal women generally exhibited a decreased BMD. However, our study demonstrated that preoperative BMD of Group A was an average 81.7% of the YAM value and that of Group B was an average 85.1% YAM value. With regard to diagnosis of primary osteoporosis in Japan, BMD should be calculated and evaluated by % YAM value compared to healthy 20 to 44-year-old adults [20]. T-score is almost identical to YAM value, but it is the standard score. Z-score is the comparison to the age-matched normal and is not used as diagnosis of osteoporosis. The diagnosis of primary osteoporosis was defined as a BMD with less than 70% measured by DXA without fragility fracture [20]. Therefore, most of the patients did not meet the criteria for osteoporosis preoperatively. Vertebral stress-shielding most likely may occur as a result of load-bearing by the fusion mass and therefore may lead to local resorption of vertebral body bone at the level of the fusion [18, 19].

Female, old age, osteoporosis, PLIF procedure, long segment fusion, and global sagittal imbalance were considered as risk factors for proximal vertebral compression fracture after posterior fusion [5, 11, 21]. Etebar et al. reported that 5 (4%) of the 125 patients who underwent spinal fusion surgery had adjacent vertebral fracture, and they concluded that postmenopausal female patients were at high risk [5]. Watanabe et al. classified the postoperative proximal junctional vertebral fractures after long instrumentation fusion into 2 types; upper instrumented vertebral fractures and supra-adjacent vertebral fractures [21]. They concluded that old age, osteopenia, preoperative comorbidities, and marked correction of severe global sagittal imbalance were risk factors of upper instrumented vertebral fracture, and that supra-adjacent vertebral fracture might be an occasional fracture that occurred naturally. However, their study sample size was only 10 cases and thus was too small to define the risk factors of postoperative vertebral fracture. Toyone et al. reported that postmenopausal female patients who underwent lumbar spinal instrumentation surgery were susceptible to develop subsequent vertebral fractures within 2 years after surgery [11]. However, they excluded patients who were on medication for osteoporosis or whose BMD was less than 80% of the YAM values. In our study, subsequent postoperative vertebral fractures within 24 months after surgery occurred in 6 cases (11.3%) in Group A and 8 cases (11.1%) in Group B, respectively. There was no significant difference between the groups within 24 months after surgery, however the rate of subsequent vertebral fracture was significantly higher in Group A than those in Group B after long follow-ups.

Hashimoto et al. reported in detail that the natural history of Japanese women with one or more vertebral fractures in their 40s, 50s, 60s, and 70s were as follows: 2.1, 10.2, 14.0, and 44.9%, respectively [22]. Postmenopausal women generally exhibited a decreased BMD and some preoperative comorbidities, and thus the possibility of incidental fracture due to aging or natural history cannot be denied. Preoperative BMD of Group A was an average 81.7% of the YAM value and most of the patients did not meet the criteria for osteoporosis preoperatively. However, postoperative BMD could deteriorate due to aging, vertebral stress-shielding, or increased stress on the adjacent unfused segments. When a BMD value exceeds a threshold of osteoporosis, subsequent vertebral fracture may occur after surgery. Our study demonstrated that patients aged 70 years and older could exceed a threshold of osteoporosis and increase in number of subsequent vertebral fractures after 24 months following posterior spinal fusion surgery.

Hart et al. reported the use of prophylactic vertebral augmentation for prevention of proximal junctional collapse cranial to multilevel fusion [23]. They suggested that elderly female patients older than 60 years undergoing lumbar instrumented fusions may benefit from prophylactic vertebroplasty or kyphoplasty. However, their study was too small (*n* = 28), and their two groups were heterogeneous and too limited in follow-up to justify their conclusion. Prophylactic vertebral augmentation for adult spinal deformity surgery might be beneficial. However, it is difficult to show that prophylactic vertebral augmentation could be beneficial for patients with degenerative lumbar pathology who undergo spinal fusion surgery for less than 4 segments without osteoporosis preoperatively.

There are several limitations in this study. First, the study was retrospective cohort design and had small number of patients included in this study. However, baseline characteristics excluding patients’ age were not statistically different between the two groups. These results seemed to reduce the confounding factors and bias about the current study. Second, there were no fully available data of presence or absence of postoperative treatment of osteoporosis. However, most of the patients did not meet the criteria for osteoporosis preoperatively. Female patients aged 70 years and older who undergo spine fusion surgery with pedicle screw fixation should take bisphosphonates postoperatively to prevent subsequent vertebral compression fractures even though they do not have osteoporosis.

## Conclusions

The incidence of subsequent vertebral fracture of female patients aged 70 years and older was significantly higher than that of the patients under 70 years of age. In the case of pedicle screw fixation in elderly female patients, it is necessary to note the high risk of subsequent vertebral fracture despite short or non-rigid fusion. Vertebral fracture after posterior fusion surgery even for degenerative lumbar pathology could occur in more than 40% of female patients aged 70 years and older. It is also important to make every effort to increase the patients’ BMD, and to take bisphosphonates postoperatively as much as possible to prevent postoperative vertebral fractures because vertebral stress-shielding may occur.

## Abbreviations

ASD: Adult spinal deformity
BMD: Bone mineral density
PJF: Proximal junctional failure
PJK: Proximal junctional kyphosis
PLF: Posterolateral fusion
PLIF: Posterior lumbar interbody fusion
YAM: Young adult mean
